# Leveraging wastewater sequencing to strengthen global public health surveillance

**DOI:** 10.1186/s44263-025-00138-w

**Published:** 2025-03-21

**Authors:** Victor Gordeev, Martin Hölzer, Daniel Desirò, Iryna V. Goraichuk, Sergey Knyazev, Helena Solo-Gabriele, Pavel Skums, Smruthi Karthikeyan, Alexandria Evans, Shelesh Agrawal, Alexander G. Lucaci, Christopher E. Mason, Justin M. Su, Cynthia Gibas, Niranjan Nagarajan, Rafael Peres da Silva, Nicolae Drabcinski, Viorel Munteanu, Lingyu Zhan, Julia Rubin, Nicholas C. Wu, Andrew Trister, Dumitru Ciorba, Viorel Bostan, Andrei Lobiuc, Mihai Covasa, Roel A. Ophoff, Alex Zelikovsky, Mihai Dimian, Serghei Mangul

**Affiliations:** 1 Department of Electrical Engineering and Computer Science, Ștefan cel Mare University of Suceava, Suceava, 720229 Romania; 2 Department of Computers, Informatics, and Microelectronics, Technical University of Moldova, Chisinau, 2045 Republic of Moldova; 3 Genome Competence Center, Robert Koch Institute, Berlin, 13353 Germany; 4 Sonoma County Public Health Laboratory, Sonoma County Department of Health Services, Santa Rosa, CA 95404 USA; 5 Department of Pathology and Laboratory Medicine, David Geffen School of Medicine, University of California, Los Angeles, Los Angeles, CA USA; 6 Department of Clinical Pharmacy, USC Alfred E. Mann School of Pharmacy and Pharmaceutical Sciences, University of Southern California, Los Angeles, CA 90033 USA; 7 Department of Chemical, Environmental, and Materials Engineering, University of Miami, Coral Gables, FL USA; 8 School of Computing, University of Connecticut, Storrs, CT USA; 9 Department of Engineering and Applied Science, California Institute of Technology, Pasadena, CA USA; 10 Center for Neurobehavioral Genetics, Semel Institute for Neuroscience and Human Behavior, University of California, Los Angeles, Los Angeles, CA USA; 11 Chair of Water and Environmental Biotechnology, Institute IWAR, Technical University of Darmstadt, Darmstadt, Germany; 12 Department of Civil and Environmental Engineering Sciences, Technical University of Darmstadt, Darmstadt, Germany; 13 Department of Physiology and Biophysics, Weill Cornell Medicine, New York, NY 10065 USA; 14 WorldQuant Initiative for Quantitative Prediction, Weill Cornell Medicine, New York, NY 10065 USA; 15 Department of Bioinformatics and Genomics, University of North Carolina at Charlotte, Charlotte, NC USA; 16 Department of Biochemistry, Yong Loo Lin School of Medicine, National University of Singapore, Singapore, 117596 Republic of Singapore; 17 Genome Institute of Singapore, Agency for Science, Technology and Research (A*STAR), Singapore, 138672 Republic of Singapore; 18 Department of Biological and Morphofunctional Sciences, College of Medicine and Biological Sciences, Ștefan cel Mare University of Suceava, Suceava, 720229 Romania; 19 The Collaboratory, Institute for Quantitative and Computational Biosciences, University of California, Los Angeles, Los Angeles, CA 90095 USA; 20 Health Data and Epidemiology Unit, Sonoma County Department of Health Services, Santa Rosa, CA 95405 USA; 21 Department of Biochemistry, University of Illinois Urbana-Champaign, Urbana, IL 61801 USA; 22 Center for Biophysics and Quantitative Biology, University of Illinois Urbana-Champaign, Urbana, IL 61801 USA; 23 Carl R. Woese Institute for Genomic Biology, University of Illinois Urbana-Champaign, Urbana, IL 61801 USA; 24 Carle Illinois College of Medicine, University of Illinois Urbana-Champaign, Urbana, IL 61801 USA; 25 Verily Life Sciences, LLC, Dallas, TX 75019 USA; 26 Department of Basic Medical Sciences, College of Osteopathic Medicine, Western University of Health Sciences, Pomona, CA 91766 USA; 27 Department of Computer Science, College of Art and Science, Georgia State University, Atlanta, GA USA; 28 Integrated Center for Research, Development and Innovation for Advanced Materials, Nanotechnologies, Manufacturing and Control Distributed Systems (MANSiD), Ștefan cel Mare University of Suceava, Suceava, 720229 Romania; 29 Department of Computers, Electronics and Automation, Ștefan cel Mare University of Suceava, Suceava, 720229 Romania

Sequencing-based wastewater surveillance offers a scalable and cost-effective approach for monitoring pathogens circulating within communities. We explore innovations and necessary actions, calling for standardized procedures for sample collection, processing, and sequencing, improved pathogen enrichment and targeted sequencing methods, and dedicated bioinformatics tools and databases to strengthen wastewater-based pathogen surveillance.

## Background

Pathogen surveillance is pivotal for mitigating the spread of infectious diseases and safeguarding public health. Conventional microbial surveillance methods include a wide array of pathogen-specific culturing techniques, molecular assays, and strain typing schemes to monitor infectious agents and disease-causing serotypes, requiring significant laboratory capacity and being difficult to scale up [1]. At the same time, clinical genomic surveillance and testing provide data on an individual basis, requiring extensive sampling of individuals to evaluate disease burden at the population level and to track circulating and emerging pathogens and their variants [2]. By contrast, wastewater genomic surveillance through sequencing and, more broadly, metagenomics-enabled microbial surveillance are scalable with regard to both the size of the sampled population and the number of monitored pathogens and do not require direct interaction with the infected individuals, thereby reducing cost and labor, while minimizing potential pathogen exposure [1, 3]. While methods such as qPCR or digital PCR provide highly sensitive detection and accurate quantification of specific pathogen DNA or RNA in wastewater, they are limited to a predefined number of targets they can evaluate [2, 4]. In comparison, sequencing using both short- and long-read technologies enables comprehensive and accurate genotyping and, potentially, target-agnostic or semi-agnostic surveillance [4]. This capability allows for the unrestricted detection of pathogens and their variants. Wastewater sequencing also allows identification of co-occurring pathogen lineages, including closely related ones, and, for certain pathogens, enables estimation of their relative prevalence within the population [2]. This is particularly significant given that different lineages can exhibit distinct phenotypic features, such as infectivity, transmissibility, and virulence, as exemplified by SARS-CoV-2 lineages during the COVID-19 pandemic.

To reliably inform public health action along with traditional methods for pathogen monitoring, wastewater surveillance must produce high-quality data, obtaining which critically depends on the design and implementation of the wet lab experiments and the quality of downstream bioinformatics analysis. To fully harness the potential of wastewater-based measurements, advancements across multiple dimensions are needed. Key areas include: developing scenario-dependent standardized procedures for sample collection, processing, and sequencing, selected for optimal performance; innovative enrichment methods to improve the recovery of pathogen nucleic acids from complex wastewater matrices, which are particularly critical for rare or low-abundance targets; and dedicated bioinformatics methods and databases specifically tailored to wastewater data.

## Standardizing sampling and processing of wastewater

Wastewater samples exhibit highly variable properties depending on the sampling site and stage during the sewage life cycle, the collection method, temperature, storage time, geographic region, and numerous other factors. This variability often necessitates longitudinal monitoring at the same location to reliably infer trends in pathogen or variant prevalence [4]. Further variation can arise from the laboratory staff and differences in protocols, equipment, and reagents used for pathogen concentration, nucleic acid extraction, library preparation, and sequencing, which can significantly affect the yield and integrity of the pathogen genetic material as well as the yield and quality of the sequencing data [4]. Given the substantial impact of these variables on downstream analysis, robust wastewater surveillance must account for and minimize sources of variation through standardized sampling methods and strategies, including the development of epidemiologically defined protocols for wastewater sampling site selection [5]. Achieving such standardization requires extensive prior assessment of the impact of numerous physico-chemical and biological factors on sampling efficiency and relevance. Additionally, identifying and adopting the best-performing sample processing methods as standards is essential, recognizing that different pathogens and surveillance scenarios will require tailored standards.

## Advances in target enrichment

Wastewater contains a complex genomic background dominated by a few commonly occurring taxa, while pathogens, by contrast, are typically present in trace quantities. Pathogens from different taxonomic groups can be concentrated or enriched using sequence-agnostic laboratory methods based on properties such as size, sedimentation coefficient, non-specific electrostatic binding, and other properties, or through RNA/DNA depletion [4]. However, the most effective methods for enriching pathogen sequences in the final data are targeted sequencing approaches [6]. While shotgun sequencing can theoretically detect any target without prior knowledge about its nature and is occasionally applied for pathogen-agnostic wastewater surveillance [7], it typically results in wasted sequencing capacity and vastly insufficient coverage for sensitive pathogen detection [4, 6]. As sequencing costs continue to decline, ultra-deep shotgun sequencing could potentially address the problem of low sensitivity by achieving near-complete characterization of the genomic diversity of wastewater samples.

In contrast to shotgun sequencing, targeted amplicon sequencing of the whole pathogen genome or specific regions ensures sufficient coverage for detecting pathogenic variants and has become the gold standard for the surveillance of certain pathogens [4, 6]. However, designing, optimizing, and validating each amplicon panel requires significant effort to achieve uniform amplification across the entire target sequence for all relevant pathogen variants. Moreover, these panels must be continuously updated, as new mutations occurring in the primer binding regions can lower amplification efficiency, resulting in variable amplicon abundance and even in the complete dropout of certain amplicons [8].

An alternative and widely used targeting approach uses hybridization-based capture probes, which can enrich target sequences by several orders of magnitude and are significantly more tolerant to potential mutations in the pathogen’s genome. Captured sequences can differ by up to 15% from known references, enabling the detection of related pathogens or variants [3]. As a downside, this method often produces a high proportion of off-target reads, especially in wastewater samples, resulting in much lower sequencing coverage of the target(s) compared to amplicon sequencing [6]. Despite their high cost, commercial hybrid capture panels, capable of covering thousands of pathogen targets, represent a major advancement in scalability for the wastewater surveillance field. Still, full genome coverage and genotyping are typically achievable only for a small number of targets, even when combined with deep sequencing [3]. Notably, both hybrid capture enrichment and especially amplicon sequencing rely on prior knowledge of target genome sequences, usually obtained through clinical sequencing. As a result, the surveillance of novel pathogens can face significant delays, particularly when timely action is critical.

A potentially transformative innovation in wastewater surveillance could be represented by adaptive sampling, a feature of Nanopore sequencing that enables selective ejection of the DNA or RNA molecule passing through the pore based on the partial sequence/signal obtained from it in real time [9]. This technology offers two enrichment strategies: positive selection for known pathogens and negative selection against irrelevant taxa in wastewater, with the latter approach having the potential to facilitate pathogen-agnostic surveillance. It is important to note that adaptive sampling has important constraints. In particular, excessive strand rejection leads to a higher probability of pore blockage and lower total sequencing yield, although flow cells can be partially restored through washing [9]. Moreover, in the context of wastewater surveillance, the heavy nucleic acid fragmentation, in particular of RNA, might prevent optimal enrichment efficiency for certain pathogens, requiring pre-filtering of the nucleic acids by fragment length using wet lab methods. Although the current level of enrichment achievable with adaptive sampling is vastly insufficient for directly analyzing low-abundant targets in wastewater metagenomes, there is significant potential for further improvements in the decision time and accuracy for the partially sequenced molecules [9]. This could be further complemented by improvements in pore chemistry to lower pore blockage rates and increase overall enrichment efficiency. By enabling enrichment during rather than before sequencing, adaptive sampling has the potential to change the paradigm in targeted sequencing, reducing reliance on wet lab-based enrichment, thereby overcoming its associated limitations and biases, while saving time, effort, and costs. Despite the current limitations, it is important to explore whether adaptive sampling might find an application in wastewater surveillance already today. For instance, combining adaptive sampling with hybrid capture-based enrichment could significantly reduce the proportion of off-target reads, increasing the coverage of pathogen genomes and pathogen detection sensitivity.

## Advancing bioinformatics methods and databases

Bioinformatics tools for taxonomic profiling of metagenomic data are usually inadequately equipped for wastewater surveillance. First, they must balance accuracy with speed when querying their large genome reference databases, which should ideally encompass all taxa expected to occur in the sampled metagenome. This lowers the target identification accuracy, decreases detection sensitivity, and complicates the differentiation of closely related pathogen variants [10]. Second, the generic genome reference databases used by taxonomic profilers do not specialize in covering the full, up-to-date spectrum of annotated variants known for many pathogens. Finally, bioinformatics methods must also account for the specifics of wastewater sequencing data, which is affected by nucleic acid degradation, fragmentation, and the limitations associated with target enrichment methods, such as PCR errors and amplicon dropout [8].

Consequently, dedicated bioinformatics methods and algorithms are needed for accurate detection and genotyping of pathogens and their variants in wastewater sequencing data. Significant effort has been invested in developing pipelines and algorithms, including the plethora of methods designed for SARS-CoV-2 variant composition estimation in wastewater [11]. However, such methods commonly focus on a single pathogen and one specialized genome reference database. The external references they use as well as their internal representations, such as the UShER-derived mutational “barcodes” used by Freyja [2], are typically stored in non-interchangeable formats, making it challenging to adapt these methods for other pathogens. To address these limitations, computational methods should be designed to additionally accept user-sourced references in a universal interchangeable format, for instance, as representative genome sequences of the corresponding pathogens and their lineages/strains. This flexibility would make the tools easily repurposable for new targets, while potentially ensuring scalability for monitoring multiple targets in a single run, such as in data obtained from hybrid capture panels.

In parallel, systematic and comprehensive benchmarking of existing bioinformatics tools for pathogen surveillance is essential to identify the best-performing methods and algorithms for different scenarios. These tests should cover data obtained from various wastewater samples, processed with different laboratory methods, enriched and sequenced with different technologies, having different read lengths, error rates, and coverage levels. To achieve this goal, a diverse collection of benchmarking datasets must be established, reflecting the variability of wastewater surveillance data. These datasets could serve as community reference standards, akin to those used in the Critical Assessment of Metagenome Interpretation competition [10]. Furthermore, it is important to identify existing bioinformatics methods that can be repurposed for other targets and use standardized data structures representing reference pathogen genomes. These tools must also account for the types of genetic variation characteristic for specific pathogens, such as mismatches, indels, and recombination events. The best-performing and universally applicable tools, or a combination of these, could form the foundation of flexible, accurate, and computationally efficient bioinformatics pipelines tailored to the unique challenges of wastewater-based pathogen surveillance.

In addition to dedicated bioinformatics methods, unified wastewater-specific pathogen genome reference databases are needed, which must cover all pathogens potentially detectable in wastewater, including known, emerging, and cryptic variants. The construction of these databases should account for the observed variation in results caused by differences in the selected genome references for the same pathogens or strains [12]. Given the lower frequency of clinical sequencing between outbreaks, these databases should also incorporate pathogen-related metagenome-assembled genomes obtained from wastewater and other relevant environments. Obtaining such genomes could be facilitated by recent advancements in large-scale genomic screening tools such as branchwater [13] and LOGAN [14], which offer significant capabilities for interrogating large metagenomic wastewater sequencing samples. For example, branchwater offers rapid comparisons between query genomic references and large metagenomic datasets by identifying sequence similarities through sketch-based methods, enabling the detection of closely related microbes, including yet-uncultured and emerging pathogens. In combination with comprehensive databases such as the NCBI Sequence Read Archive (SRA) or the LOGAN collection of petabyte-scale sequencing data assemblies, these tools help place novel sequences in a broader evolutionary context and identify ‘missing links’ or add previously undefined genomic context, providing high sensitivity in identifying organisms with low prevalence that may otherwise go undetected. Additionally, with the advent of large language models, AI-driven tools can facilitate the integration of diverse databases, enabling the development of generalizable representations that can be flexibly adapted to different settings, thereby enhancing bioinformatics tools while minimizing the need for manual data curation [15]. Such approaches can improve pathogen detection and the ability to monitor cluster dynamics, evolution, and potential reservoirs in wastewater ecosystems, providing researchers with crucial genomic information for advanced pathogen surveillance and public health monitoring.

## Conclusions

Leveraging the full potential of wastewater surveillance for microbial pathogens necessitates the development of optimized scenario-dependent protocols for wastewater sampling, standardized sample processing methods, and advancements in enrichment techniques for pathogen DNA/RNA. Additionally, there is a need for dedicated reference genome databases for pathogens detectable in wastewater as well as scalable bioinformatics methods to accommodate diverse pathogens and surveillance scenarios effectively.

## Data Availability

No datasets were generated or analysed during the current study.
